# Visuomotor Dissociation in Cerebral Scaling of Size

**DOI:** 10.1371/journal.pone.0151484

**Published:** 2016-03-10

**Authors:** Adriaan R. E. Potgieser, Bauke M. de Jong

**Affiliations:** 1 Department of Neurosurgery, University Medical Center Groningen, University of Groningen, Groningen, The Netherlands; 2 Neuroimaging center, University Medical Center, University of Groningen, Groningen, The Netherlands; 3 Department of Neurology, University Medical Center Groningen, University of Groningen, Groningen, The Netherlands; Centre de Neuroscience Cognitive, FRANCE

## Abstract

Estimating size and distance is crucial in effective visuomotor control. The concept of an internal coordinate system implies that visual and motor size parameters are scaled onto a common template. To dissociate perceptual and motor components in such scaling, we performed an fMRI experiment in which 16 right-handed subjects copied geometric figures while the result of drawing remained out of sight. Either the size of the example figure varied while maintaining a constant size of drawing (visual incongruity) or the size of the examples remained constant while subjects were instructed to make changes in size (motor incongruity). These incongruent were compared to congruent conditions. Statistical Parametric Mapping (SPM8) revealed brain activations related to size incongruity in the dorsolateral prefrontal and inferior parietal cortex, pre-SMA / anterior cingulate and anterior insula, dominant in the right hemisphere. This pattern represented simultaneous use of a ‘resized’ virtual template and actual picture information requiring spatial working memory, early-stage attention shifting and inhibitory control. Activations were strongest in motor incongruity while right pre-dorsal premotor activation specifically occurred in this condition. Visual incongruity additionally relied on a ventral visual pathway. Left ventral premotor activation occurred in all variably sized drawing while constant visuomotor size, compared to congruent size variation, uniquely activated the lateral occipital cortex additional to superior parietal regions. These results highlight size as a fundamental parameter in both general hand movement and movement guided by objects perceived in the context of surrounding 3D space.

## Introduction

Grasping an object heavily relies on parietal—premotor circuitry [1–3] within which two basic processing streams can be discerned. While superior parietal and dorsal premotor cortex (PMd) particularly subserve aligning the direction of movement with the target's spatial location [2,4–6], antero-inferior parietal and ventral premotor cortex (PMv) are stronger implicated in linking object shape and prehension [1,7,8]. Interrelated with the latter, visual object identification along an occipito-temporal processing stream [9] is based on building an image from elementary shapes, while recognizing a graspable object requires existing knowledge about its qualities [10–12]. In this, object identity remains constant, despite changing its image by viewing it from different perspectives. Similarly, the inferred size of an object remains constant when the size of the retinal image varies by placing it at different distances.

In grasping, changes in object perspective or distance require adjusted visuomotor transformations. In this, the perceived distance is inferred from the spatial relationships of such an object within its 3D environment, which enables scaling the hand aperture to the actual size of the target [13,14]. These variables in natural circumstances thus imply elaborate visuomotor transformations beyond simple matching visual shape and prehension, which fit the model describing that visuomotor transformations are mediated by internal spatial reference frames for optimal alignment of parameters derived from the modalities involved [15]. In the present study, we aimed to explore cerebral circuitry involved in these more complex aspects of achieving visuomotor congruity, particularly those concerning scaling of size. To address this specific issue of scaling visual size, we employed a drawing task in which sizes of visually presented figures and drawn copies varied independently from each other.

Studies that addressed visual congruity have not only advanced understanding of visual perception, but also provided models for mechanisms underlying visuomotor control. In the classical experiment of Shepard and Metzler, the time it took to assess whether pairs of 3D object pictures portrayed in different orientations concerned the same object or not, appeared to increase linearly with the angle of incongruity [16]. Although this experiment was designed for perceptual assessment, the results laid ground for the concept of mental manipulation ('mental rotation') of one of the two objects. The involvement of such covert motor function gained support from functional brain imaging [17,18], revealing a consistent role of the right parietal cortex [19]. The assessment of visual incongruity with an even stronger aspect of (covert) motor function was addressed in functional imaging studies that employed incongruity in hand positions [20,21], hand movements [22] or tool positions [23], demonstrating the involvement of the PMd, particularly in the left hemisphere and often associated with left parietal activation. These imaging results thus suggest a distinction in solving incongruity dominated by either perception or action, associated with right and left hemisphere functions, respectively. In an overt visuomotor experiment, we previously demonstrated such dissociation in visuomotor transformations concerning spatial incongruity between the axial orientations in displayed zigzag lines and the directions of drawing them [24]. In this, activations of the right parietal cortex and right PMd were related with visual incongruity while left PMd activation was particularly seen in motor incongruity, further supporting the model of an internally defined coordinate system onto which visual and motor coordinates are separately aligned [15].

A challenge to such internal coordinate system also occurs when estimating the actual size of a visually perceived object placed at varying distances in its environment. From such context information one may infer whether an object is graspable or not. The latter implies using a body-centered coordinate frame for referencing the range of the hand aperture. The environmental context of an object influences the perception of its size [25]. Viewing an image in which this object-environment relation is artificially manipulated may even induce illusionary disproportions of object size [25–27], which underscores the dynamic character of coordinate frames that anchor a representation of the visual world. An intriguing observation further highlighting that size is a specific parameter in visuomotor coordination concerns copying a written text by handwriting. In normal healthy subjects, the resulting text has a regular pattern constituted by letters of similar size. Although the script of patients suffering from Parkinson's disease tends to become smaller during writing (micrographia) [28], this size is relatively enlarged when they copy the text without seeing their own text [29]. This similarly holds for copying zigzag figures without visual feedback [24].

In the present functional Magnetic Resonance Imaging (fMRI) experiment we employed a visuomotor paradigm essentially characterized by copying elementary figures without visual feedback upon drawing. In two conditions with visuomotor congruity, copies were made of figures with the same or variable sizes. Two conditions with visuomotor incongruity enabled us to separately challenge, and thus dissociate visual and motor aspects of the underlying visuomotor transformations. This was achieved by either (i) varying size of the example figure while maintaining a constant size of drawing or (ii) keeping the size of the examples constant while subjects were instructed to make copies either twice as large or half in size. We hypothesized that, compared to copying with congruent size, particularly right superior parietal and PMd activations would occur in copying with visual template variation ('visual incongruity') and that the left PMd would show stronger activation in relation with motor variation ('motor incongruity'). Such dissociation would support the concept that size is a fundamental parameter, used by the brain in a similar way as spatial orientation and direction to encode visuomotor transformations. By employing figure drawing with a pencil as a motor task, and not e.g. grasping movements, we were able to pinpoint on visual and motor parameters, avoiding the introduction of proprioceptive variation implicated in tuning the hand's aperture to the observed figure size.

## Materials and Methods

### Subjects

Sixteen healthy adult right-handed volunteers (eight female), mean age 25.5 years (SD 2.8 years), participated in this study [30]. All had Dutch as a native language. The Edinburgh Handedness Inventory [31] confirmed that all subjects were right-handed with scores that varied between 60 and 100 (mean 90.0, SD 12.8). Subjects had no neurological or psychiatric disorders and did not suffer from lesions of the upper extremities. This study was approved by the Medical Ethics Committee of the University Medical Center Groningen. All subjects signed an informed consent. Study procedures were explained and practiced briefly immediately before the experiment until subjects understood the tasks.

### Experimental procedure

**Fig 1:**
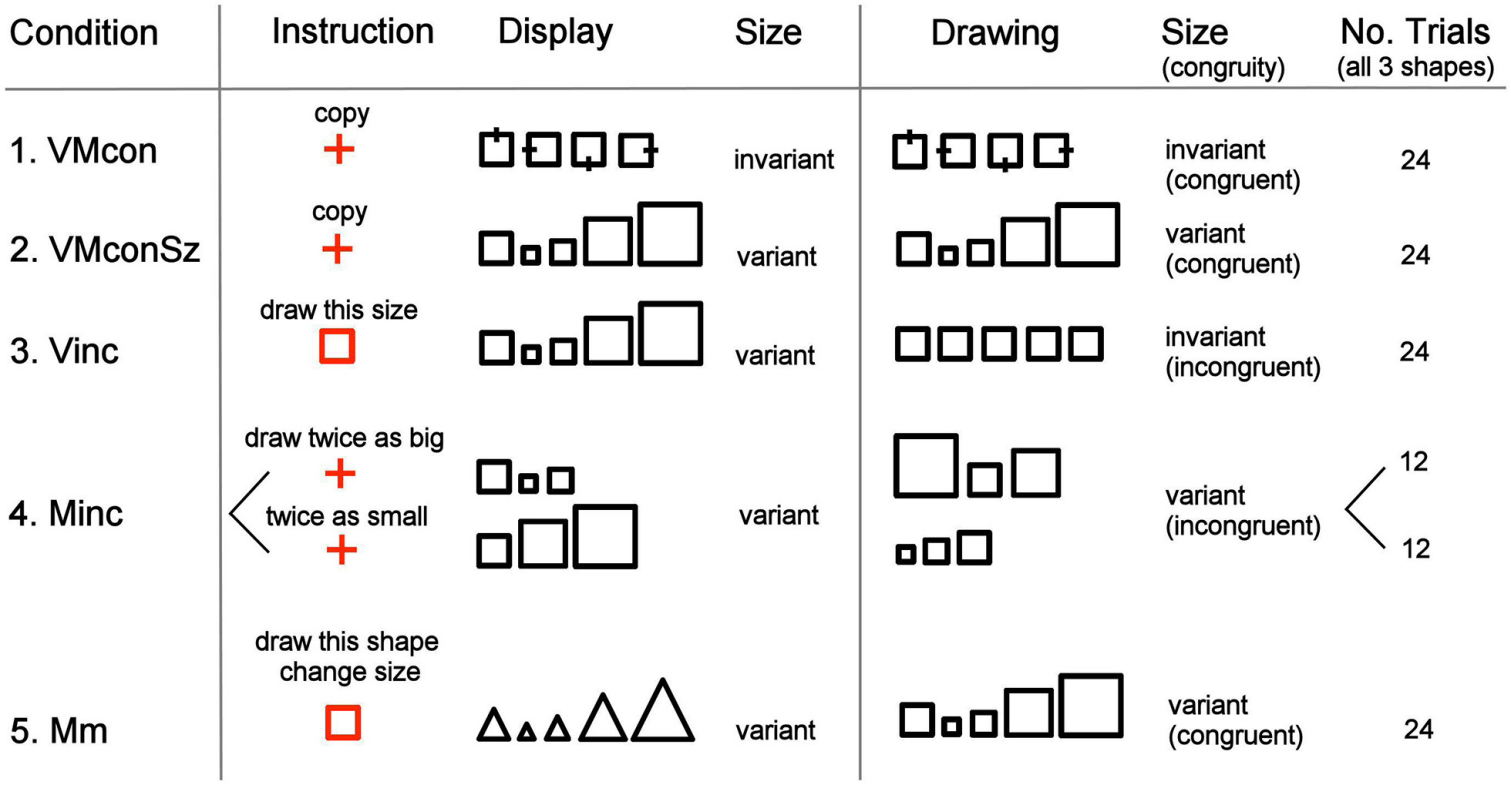
Schematic overview of the experimental conditions. Tasks were performed in 18 s trials, each consisting of 6 successive figure displays that had to be copied as instructed by the preceding text (see also Fig 2). To optimally illustrate the balanced design concerning size of the displayed and drawn figures, the five possible sizes are depicted in a structured order for one of the three figure shapes (square), while such order was pseudo-randomized within the experimental trials. The illustated size distribution similarly held for the other two figures, i.e. the triangle and rhombus. VMcon = visuomotor congruence, VMconSz = visuomotor congruence with different sizes, Vinc = visual incongruence, Minc = motor incongruence, Mm = motor memory.

Subjects were positioned in the scanner with pillows under their flexed knee, providing stable support for drawing on a metal-free drawing-case placed on their lap. They viewed a monitor screen on which instructions and visual stimuli were displayed using ‘Presentation’ (Neurobehavioural systems, Inc. Albany, USA). The paradigm was constituted by five stimulus-response conditions and a baseline condition of viewing a central fixation cross on a monitor screen (see overview in Fig 1). In the visuomotor conditions, one of three geometrical figures was presented on the screen, i.e. either a square, triangle or rhombus, while subjects had to draw figures with a pencil in their right hand. They had no visual feedback on their performance, which enabled us to specifically study the effect of size scaling in visuomotor transformation not confounded by corrections due to visual feedback. In conditions 1–4, successively displayed figures had the same geometrical shape while the displayed size varied in conditions 2, 3 and 4. In conditions 1 and 2, subjects exactly copied the displayed figures, which implied that the size of drawing was invariant in condition 1 and varied in condition 2. In condition 3, each figure shape (which had variable sizes) had to be copied by drawing with invariant size, identical to the initial reference figure. In condition 4, the presented figures (with invariant size) were copied larger or smaller. In condition 5, both shape and size of the presented figure varied within a trial while subjects had to draw the initially presented figure shape with size of the actually presented figure. The task instruction for a trial was specified by a short text on the screen, presented during 2.5 s before each trial. In such following 18 s lasting trial, a series of six figures was presented (3 s per figure).

In conditions 1, 2 and 4, the instruction text was placed above a central cross, while in conditions 3 and 5 the text was accompanied by a reference figure. In condition 1 (visuomotor congruence, VMcon), subjects had to copy the presented figures, which all had the same size. In this condition, a small bar was placed perpendicular to one of the figure edges to realize variation between the successive figures, serving a maintained level of attention. This was required because the presented figures would otherwise have been identical in condition 1, while in the other conditions successive images differed. In condition 2 (visuomotor congruence with different sizes, VMconSz), the figures varied in size and had to be copied with corresponding variation. Within the series of six presented figures, size was either 0.5, 0.75, identical, 1.5 or double to the reference figure. In condition 3 (visual incongruence, Vinc), presentations were with size variation similar to condition 2, while now subjects had to copy the figure shape with an invariant size that matched the size of the reference figure that accompanied the instruction. In condition 4 (motor incongruence, Minc), subjects were instructed to copy the presented figure twice as large in half of the trials or two times smaller in the other trials of this condition. In this condition, size of the presented figures varied similar to the variations in conditions 2 and 3, but the figures were arranged in such a way that in the trials of enlarged copying, example sizes 1.5 and 2 were excluded while in the trials of making smaller copies, figure sizes 0.75 and 0.5 were not presented. Condition 5 (motor memory, Mm) was added to balance possible memory effects expected in condition 3 (Vinc). In this visuomotor condition, subjects had to copy the reference figure but with variable sizes. This size was indicated by the size of the six successively presented figures of which the shape differed from that of the trial's reference figure. The specific instruction text (in Dutch) that accompanied either the central cross or the reference figure was ‘copy’ for conditions 1 and 2, ‘draw this size’ for condition 3, ‘draw twice as big' or 'draw twice as small’ for condition 4 and ‘draw this shape, change size’ for condition 5. Instruction for the baseline condition was given by 'fixate' with the central cross beneath it. The instruction for the latter was followed by five repeated presentations of the fixation cross, lasting 3 s each, and briefly interrupted by a 50 ms blank screen, similar to the switching of figures.

**Fig 2:**
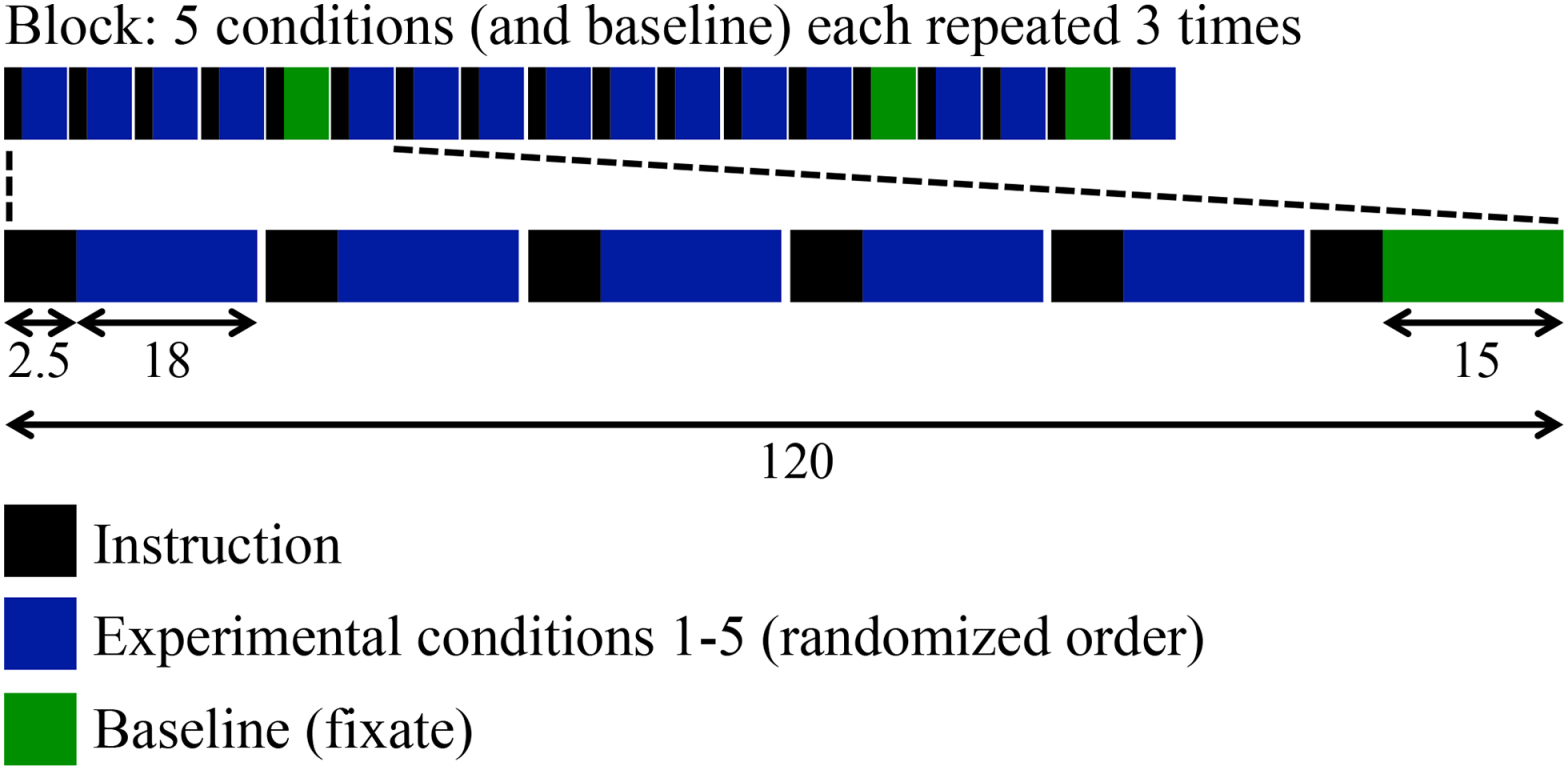
Experimental paradigm, overview of data acquisition. Subjects performed five different task conditions and one baseline condition. Conditions were presented in a pseudorandomized order. The entire experiment consisted of eight different blocks, divided over two runs. The indicated time scale is in seconds. Each condition was thus presented 24 times.

Visual stimuli were presented in a block design, with eight different blocks equally divided over two runs (see Fig 2). In each block, trials of the five stimulus-response conditions were each presented three times, while the fixation baseline was similarly presented three times. In this way every condition was repeated 24 times. The conditions were presented in a pseudo-randomized interleaved order to avoid confounds due to ordering effects. Subjects were in the scanner for about 50 minutes. Between the two runs, a T1-weighted anatomical image was acquired and a new paper was placed on the drawing-case. This implied that for each run, drawings were made at the same spot on the paper.

### Data acquisition

Data acquisition was performed using a 3 T Philips MR system (Best, The Netherlands) with a standard 32-channel SENSE head coil. Functional images were acquired using a gradient-echo T2* Blood Oxygen Level Dependent (BOLD) technique using the following parameters: field of view 224 x 136.5 x 224 mm, TR = 2000 ms, TE = 28.0 ms, flip angle 70°, 39 slices without slice gap, isotropic voxels 3.5 x 3.5 x 3.5 mm, axial orientation, 721 volumes per run. A T1-weighted 3D anatomical scan was acquired to obtain high-resolution anatomical information with a field of view of 232 x 170 x 256 mm, TR = 9.0 ms, TE = 3.5 ms, flip angle 8°, 170 slices without slice gap, voxel size 0.9 x 1.0 x 1.0 mm.

### Data analysis

Image processing and voxel-based statistical analysis was conducted using Statistical Parametric Mapping [32], version 8 (2009, Wellcome Department of Cognitive Neurology, London, UK: http://www.fil.ion.ucl.ac.uk/spm). Preprocessing with SPM included realignment, coregistration with the high-resolution T1 anatomical image, normalization to the Echo Planar Image (EPI) of the Montreal Neurological Institute (MNI) brain and smoothing with a Gaussian filter of 8 mm full width at half maximum (FWHM).

Cerebral activations were rendered onto the standard MNI brain. All five experimental conditions were modeled in a block design at subject level for statistical analysis of regional differences in cerebral activations, in which the baseline (rest) condition with the fixation cross was implicitly modeled. We included regressors describing head motion. These included three rotational and three linear movement parameters together with their quadratic, as well as the derivatives of these computations. After that, the subject-level contrasts were analyzed at group level using one-way repeated measurements ANOVA (random effect analysis). Minc and Vinc were each contrasted to VMcon and VMconSz, as well as to each other, while the two congruent conditions were also compared with each other. The resulting set of voxel values for the assessed contrasts constituted the associated SPM of the t-statistics (SPM<T>) and were thresholded at initial voxel response height p<0.001 with extent threshold k = 8 voxels. Resulting clusters of increased activation were considered statistically significant at cluster-level p<0.05, corrected for the entire brain volume (FDR-corrected). In order to provide optimal insight in the coherent data set obtained from the various conditions, the results are displayed at p<0.001 uncorrected voxel-level significance in the figures, while the corresponding cluster-level corrected activations are reported in the tables.

## Results

**Fig 3:**
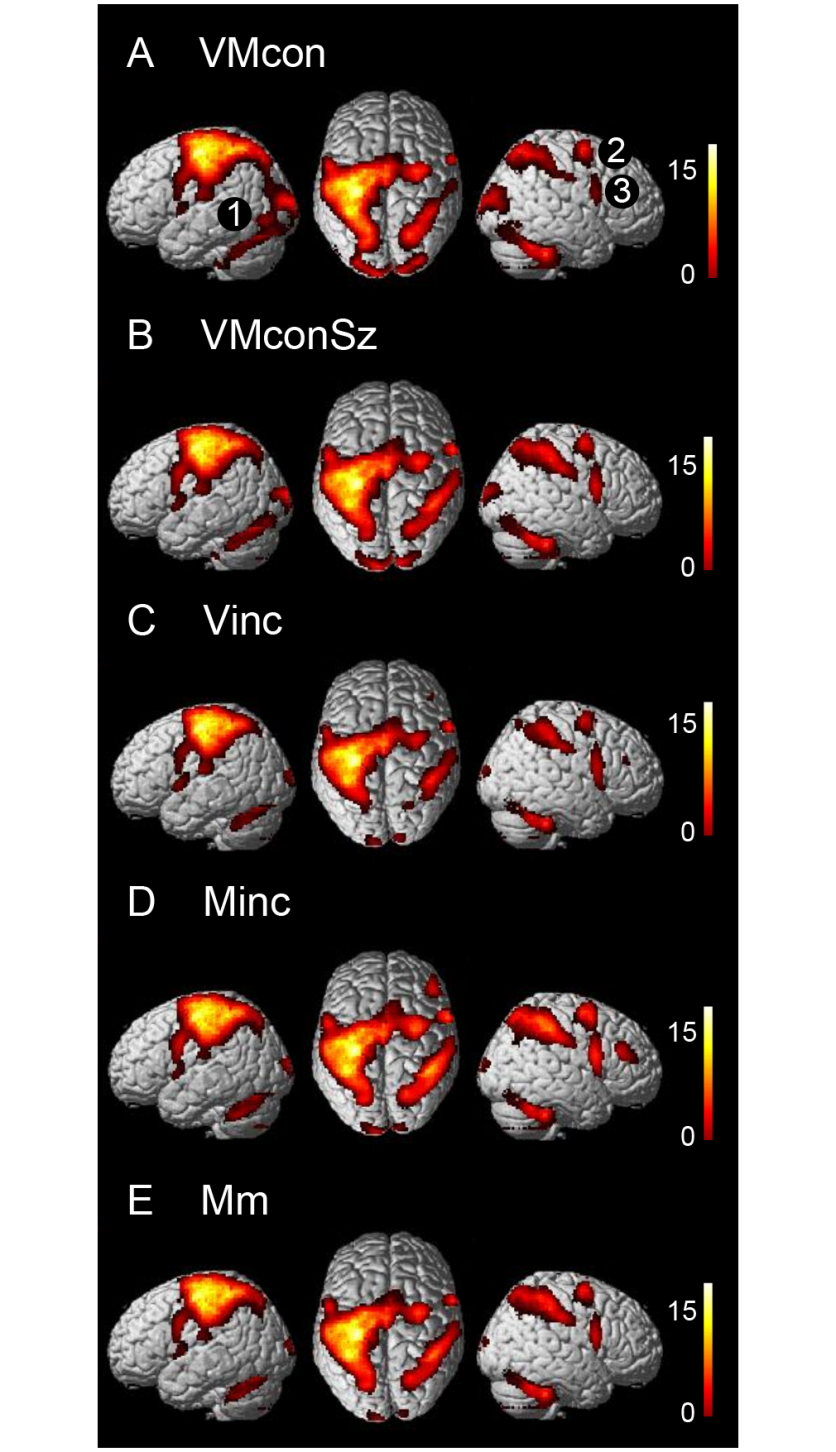
Task-related cerebral activations compared to baseline. (A) VMcon, (B) VMconSz, (C) Vinc, (D) Minc, (E) Mm. The presented activations resulted from analyses using a statistical threshold of p<0.001 uncorrected, with an extended voxel threshold (k) of 8 voxels. Clusters are rendered onto the surface of a standard anatomical brain volume (Montreal Neurological Institute, MNI). The color bars represent T-values. VMcon = visuomotor congruence, VMconSz = visuomotor congruence with different sizes, Vinc = visual incongruence, Minc = motor incongruence, Mm = motor memory. 1 = lateral occipital cortex, 2 = dorsal premotor cortex, 3 = ventral premotor cortex.

Relative to the baseline of viewing the central fixation cross, the patterns of cerebral activations related to the five visuomotor conditions were robust with considerable overlap (Fig 3). Common activations included the left primary sensorimotor cortex, bilateral superior and inferior parietal cortex as well as premotor regions, both PMd and PMv. In the left hemisphere, additional cortical activations were seen in the middle segment of the insula [x-40, y-2, z8] and the parietal operculum [x-48, y-24, z20]. Common subcortical activations included the posterior segment of the left putamen, extending into the pallidum towards the left thalamus [x-16, y-18, z-6]. In the cerebellum, activations were seen in the right anterior and bilateral posterior lobes.

A first impression of differences between the experimental conditions, inferred from the comparison with baseline, concerned activation of the right dorsolateral prefrontal cortex (dlPFC) which was only observed during Minc and, although to a lesser extent, Vinc. The absence of this prefrontal and additional activations in the Mm condition suggested that a working memory component was balanced among the experimental conditions (Fig 3E). This was further underscored by the fact that no Mm-related activation increase was seen in the dlPFC and inferior parietal cortex when Mm was contrasted with Vinc and Minc, respectively. The visuomotor congruity tasks VMcon and VMconSz showed additional activations that were more widely spread over the occipital cortex. In this, VMcon was the only condition with activation of the lateral occipital cortex (LOC).

With regard to the behavioral performance, camera monitoring in the scanner room confirmed that all subjects performed a drawing task when instructed to do so. The resulting drawings further provided a fair indication of this performance. For each run, however, drawings were made on the same spot of a paper, which was only replaced between the two runs. This implied that in the superimposed drawings only global contours of the condition-specific drawings could be discerned. In this way, the drawings could not be used for quantitative assessment of subject's accuracy.

### Visuomotor incongruity compared with congruity

**Fig 4:**
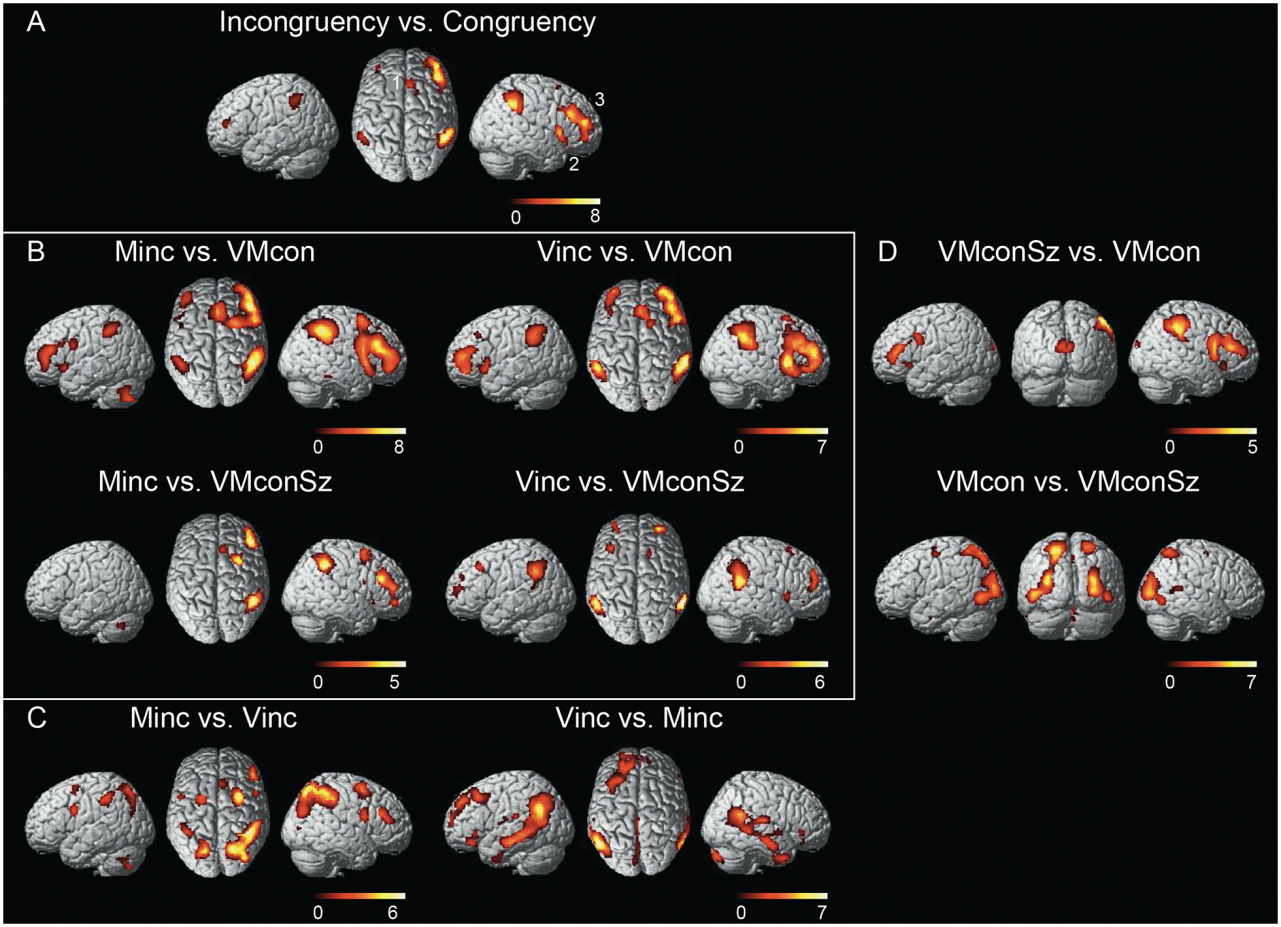
Cerebral activations revealed by contrasting the various conditions. (A) Incongruity versus congruity (Minc and Vinc contrasted with VMconSz and VMcon, (B) Minc and Vinc contrasted with VMcon and VMconSz, respectively, (C) Comparison between the incongruent conditions, (D) Comparison between the congruent conditions. The presented activations in (A) resulted from analyses using a statistical threshold of p<0.05, FWE corrected, with an extended voxel threshold (k) of 8 voxels. For the other comparisons, all activations that resulted from a statistical threshold of p<0.001 uncorrected (with k of 8 voxels) are displayed. Clusters are rendered onto the surface of a standard anatomical brain volume (Montreal Neurological Institute, MNI). The color bars represent T-values. Coordinates and T-values of significant activations after cluster-level correction are reported in Tables 1–3. VMcon = visuomotor congruence, VMconSz = visuomotor congruence with different sizes, Vinc = visual incongruence, Minc = motor incongruence. 1 = pre-supplementary motor area, 2 = anterior insula, 3 = dorsolateral prefrontal cortex.

An initial comparison of the two incongruity tasks Minc and Vinc with the congruity tasks VMcon and VMconSz revealed significant activations (p<0.05, FWE corrected for the entire brain volume) on the lateral surface of the inferior parietal cortex, bilaterally, the middle frontal gyrus (dlPFC), predominantly in the right hemisphere, the right frontal operculum extending into the right anterior insula and the right pre-SMA/dorsal anterior cingulate cortex (dACC) (Fig 4A). As will be shown by the following comparisons, effects in e.g. the inferior parietal and dorsolateral prefrontal regions were generally stronger in Minc than in Vinc. Moreover, in the two congruity tasks, variance in size (VMconSz) appeared to be a characteristic parameter that was also associated with increased responses in these parietal-prefrontal regions, relative to VMcon.

Minc contrasted to VMcon showed a pattern that included the activations described for common incongruity in the previous paragraph. In addition, increased activations were seen in the PMv bilaterally, the rostral segment of the right PMd and the anterior insula on the left side (Fig 4B). The right frontal operculum activation identified in general incongruity, now not only extended into the right anterior insula but also over the lateral aspect of the inferior frontal gyrus towards the PMv. Finally, a significant cluster was seen in the left posterior part of the cerebellum. Coordinates of significant activations (p<0.05, FDR cluster-corrected) are reported in Table 1.

**Table 1:** Cerebral activations related to Minc versus VMcon, Minc versus VMconSz and Minc versus Vinc.

Brain region (BA)	Left				Right			
	x	y	z	T-value	x	y	z	T-value
*Minc vs*. *VMcon*								
Middle frontal gyrus (45)	-42	44	8	5.2	46	42	18	8.8
	-40	40	22	5.0	46	32	28	8.5
Pre-SMA/anterior cingulate (8)					8	24	44	7.7
PMd (6)					26	8	52	5.4
PMv (6)	-44	2	28	5.3	48	6	24	7.5
Anterior insula (47)	-36	18	-4	5.1	36	24	-8	5.9
Inferior parietal cortex (40)	-52	-48	54	5.0	54	-46	54	8.7
	-52	-40	44	4.2	44	-46	42	7.8
Cerebellum	-36	-68	-42	5.2				
*Minc vs*. *VMconSz*								
Middle frontal gyrus (45)					46	32	28	5.8
					46	42	18	5.1
					34	46	10	4.2
PMd (6)					24	8	50	4.6
Inferior parietal cortex (40)					54	-48	54	5.7
					44	-50	42	5.1
*Minc vs*. *Vinc*								
Middle frontal gyrus (45)					50	32	28	5.3
					46	42	18	4.9
Pre-SMA/anterior cingulate (8)					8	20	48	4.1*
PMd (6)	-22	0	54	5.5	28	4	52	6.5
PMv (6)	-46	4	30	4.7*	48	6	22	5.1
Superior parietal cortex (7)	-20	-68	54	5.4	20	-72	62	6.6
	-24	-72	42	5.3				
Inferior parietal cortex (40)	-40	-40	42	5.8	42	-42	44	6.3
					30	-46	44	6.3
Cerebellum	-36	-64	-38	3.9*				

The MNI-coordinates and T-values of local maxima within significant clusters are reported. Initial threshold was set at voxel-level p<0.001 uncorrected, with an extended voxel threshold (k) of 8 voxels. Clusters that survived correction for the whole brain volume (p<0.05, FDR-corrected) were considered statistically significant. In addition, we report regions (*) that reached an uncorrected cluster-level significance (p<0.05). Positive x, y and z coordinates indicate respectively coordinates right, anterior and superior of the middle of the anterior commissure. VMcon = visuomotor congruence. VMconSz = visuomotor congruence with different sizes. Minc = motor incongruence. Vinc = visual incongruence. BA = Brodmann area. MNI = Montreal Neurological Institute. PMd = dorsal premotor cortex. PMv = ventral premotor cortex. pre-SMA = pre-supplementary motor area.

**Fig 5:**
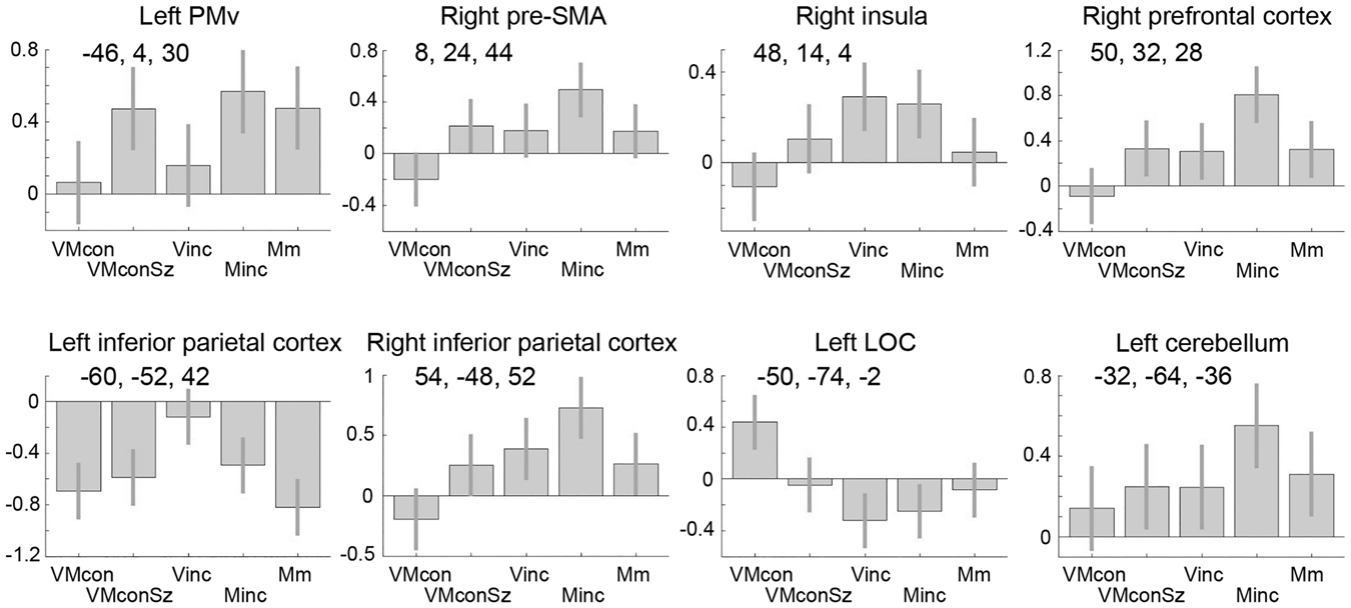
Effects of interest at foci of maximum activation relevant to size scaling. Contrast estimates are given with 90% confidence intervals. The regions of activation are reported in Tables 1 and 2, only LOC is listed in Table 3. Positive co-ordinate values for (x, y, z) refer to respectively superior, right and anterior positions (in mm) to the middle of the anterior commissure. VMcon = visuomotor congruence, VMconSz = visuomotor congruence with different sizes, Minc = motor incongruence, Vinc = visual incongruence, PMv = ventral premotor cortex, LOC = lateral occipital cortex.

**Fig 6:**
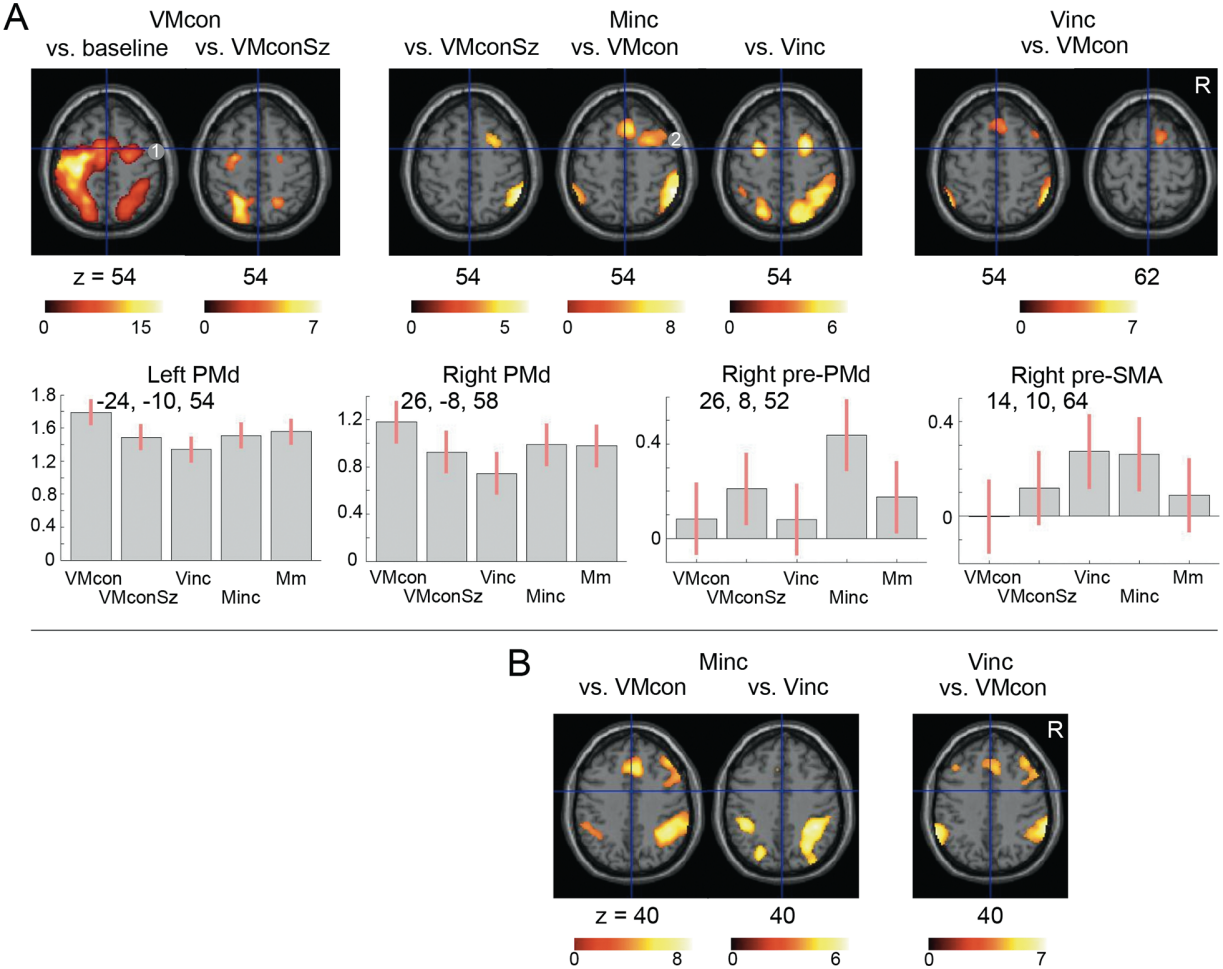
(A) Regional differentiation within the dorsal premotor cortex. Contrast estimates with 90% confidence intervals are plotted for the indicated foci of maximum activation. (B) Regional differentiation within the inferior parietal cortex for Minc and Vinc. Regional cerebral activations (p<0.001 voxel-level uncorrected, extended voxel threshold (k) 8) are projected on transversal sections of a standard anatomical brain (MNI). The z coordinate indicates the distance to the plane traversing the anterior-posterior commissures in mm. The right side corresponds to the right side of the brain. Positive co-ordinate values for x and y refer to respectively right and anterior positions (mm) to the middle of the anterior commissure. The color bars represent T-values. Coordinates of the displayed regional activations that reached statistical significance after cluster-level correction are reported in Tables 1 and 2. VMcon = visuomotor congruence, VMconSz = visuomotor congruence with different sizes, Minc = motor incongruence, Vinc = visual incongruence. 1 = dorsal premotor cortex, 2 = pre-dorsal premotor cortex.

When contrasted to VMconSz, the Minc-related activations in the left hemisphere disappeared, pointing at the important effect of changing size with maintained visuomotor congruity. The latter will be elaborated later. The activation increases that resulted from this comparison now only reached statistical significance (p<0.05, FDR cluster-corrected) in the inferior parietal cortex, rostral PMd and dlPFC of the right hemisphere (Fig 4B, Table 1). At relaxed threshold, increased right pre-SMA activation related to Minc [x8, y22, z48] (T = 4.0, p = 0.15, FDR cluster-corrected) was found (Fig 4B). The more specific involvement of these right hemisphere regions in Minc is further illustrated by the profile of plotted condition effects in these regions (Figs 5 and 6A).

While Minc was designed to identify 'action' dominance in solving visuomotor incongruity, Vinc was considered to emphasize 'perceptual' dominance by keeping the size of the drawn picture identical to the initial example while the size of the subsequent examples varied. Although the pattern of activations related to Vinc showed a resemblance with that of Minc, either compared to VMcon or to VMconSz, characteristic differences were present. Within the right dlPFC cluster, the Vinc-related focus of maximum activation was located more anterior than the Minc-related maximum, while the inferior parietal maximum was at a more ventral position (Fig 4B, Table 2). At this ventral location, the Vinc-related activation remained at the parietal surface, while the activation pattern related to Minc followed the postcentral sulcus into the horizontal limb of the intraparietal sulcus (Fig 6A and 6B). No increased PMv activation was seen in Vinc compared to the congruity tasks. On the contrary, PMv activation was even stronger in VMconSz than in Vinc (Fig 5).

**Table 2:** Cerebral activations related to Vinc versus VMcon, Vinc versus VMconSz and Vinc versus Minc.

Brain region (BA)	Left				Right			
	x	y	z	T-value	x	y	z	T-value
*Vinc vs*. *VMcon*								
Middle frontal gyrus (45)	-34	46	10	5.3	38	52	16	6.1
Pre-SMA/anterior cingulate (8)					10	24	44	4.6
					14	10	64	4.4
Anterior insula (47)	-38	18	-6	4.2	48	14	4	6.6
Inferior parietal cortex (40)	-58	-52	46	7.1	64	-42	44	7.3
Corpus callosum	-18	-44	16	5.5	20	-42	16	4.4
	-8	-24	28	5.0	8	-24	26	4.3
*Vinc vs*. *VMconSz*								
Middle frontal gyrus (45)					30	52	34	4.9
Inferior parietal cortex (40)	-60	-52	42	6.7	62	-50	42	6.0
Corpus callosum	-26	-50	18	4.6*				
*Vinc vs*. *Minc*								
Middle frontal gyrus (45)	-36	24	44	4.8				
Inferior frontal gyrus (47)	-30	34	-14	5.4*				
Superior frontal gyrus (9)	-12	48	38	4.6				
Inferior parietal cortex (40)	-54	-60	32	7.7	60	-64	20	6.3
Corpus callosum	-8	-18	30	5.7	36	-50	6	5.0
Middle temporal gyrus (21)	-60	-20	-14	5.3				
Temporal pole (20)					46	2	-38	4.4
Cerebellum					38	-84	-40	4.9

The MNI-coordinates and T-values of local maxima within significant clusters are reported. Initial threshold was set at voxel-level p<0.001 uncorrected, with an extended voxel threshold (k) of 8 voxels. Clusters that survived correction for the whole brain volume (p<0.05, FDR-corrected) were considered statistically significant. In addition, we report regions (*) that reached an uncorrected cluster-level significance (p<0.05). Positive x, y and z coordinates indicate respectively coordinates right, anterior and superior of the middle of the anterior commissure. VMcon = visuomotor congruence. VMconSz = visuomotor congruence with different sizes. Minc = motor incongruence. Vinc = visual incongruence. BA = Brodmann area. MNI = Montreal Neurological Institute. pre-SMA = pre-supplementary motor area.

### Comparisons between motor and visual incongruity

A direct comparison of Minc and Vinc underscored the bilateral involvement of the rostral PMd and PMv in specifically Minc, as well as the dorsal-ventral dissociation in the inferior parietal activations related to Minc and Vinc, respectively (Fig 4C, Table 1). Activations in the anterior insula were balanced. Actually, particularly the right anterior insula was one of the two regions that showed the same magnitude of increase in Minc and Vinc relative to the other conditions (Fig 5). The most superior part of the right pre-SMA cluster, extending over the dorsal aspect of the superior frontal gyrus [x14, y10, z64] had a similar profile (Fig 6A). The comparison of Vinc with Minc further revealed bilateral activation increases at the temporoparietal junction, involving the angular and supramarginal gyri. This pattern spread along the middle temporal gyrus towards the temporal poles (Fig 4C), although the left temporal pole cluster did not reach corrected cluster-level significance [x-48, y-6, z-36] (p = 0.27). Given (i) the profile of negative effect sizes in these ventral parietal-temporal regions, mimicking that of the left inferior parietal cortex (Fig 5), and (ii) the absence of activation increases in Vinc contrasted to baseline, provides an argument to infer that these cortical regions were particularly characterized by a relative reduction of activation in the visuomotor tasks, which was less pronounced in Vinc.

### Visuomotor congruity with variable size

Although the two congruity conditions primarily served as controls for the incongruity tasks, the effects of size variation and size constancy in the congruity tasks provided important information. Using VMconSz as control task for Minc already indicated the common involvement of the left frontal and inferior parietal cortex in these conditions. For the right hemisphere, dlPFC and inferior parietal activations related to Minc were unmistakably increased compared to the other conditions, including VMconSz (Fig 5). However, VMconSz-related activations in these regions were significantly stronger (p<0.05, FDR cluster-corrected) than in VMcon (Figs 4D and 5, Table 3). This was similarly the case for the right pre-SMA / anterior cingulate. Although activation increases were also seen in the anterior insula (Fig 4D), these clusters did not reach corrected cluster-level significance: right insula [x38, y26, z-8] (p = 0.15, FDR cluster-corrected) and left insula [x-34, y24, z-2] (p = 0.14). A most characteristic profile of increased activations was seen in the left PMv with effect sizes that were the same in those conditions that included variation in the size of drawing (Fig 5), relative to VMcon and Vinc in which the size of the drawn pictures was the same.

**Table 3:** Cerebral activations related to VMcon versus VMconSz and VMconSz versus VMcon.

Brain region (BA)	Left				Right			
	x	y	z	T-value	x	y	z	T-value
*VMconSz vs*. *VMcon*								
Middle frontal gyrus (45)	-42	42	8	4.2	48	42	18	5.2
Pre-SMA/anterior cingulate (8)					8	24	44	5.0
PMv (6)	-44	4	30	4.6	48	6	24	5.1
Inferior parietal cortex (40)					52	-34	58	5.0
					46	-40	42	4.5
Cuneus (18)					4	-94	20	4.9
*VMcon vs*. *VMconSz*								
PMd (6)	-24	-10	54	4.9				
Superior parietal cortex (7)	-22	-56	58	7.3	24	-62	62	5.4
	-20	-76	48	5.2				
Precuneus (7)					6	-54	46	4.3
Dorsolateral visual cortex (18)	-30	-90	16	5.7	32	-92	20	6.9
Lateral occipital cortex (19)	-50	-74	-2	5.8	50	-74	-4	4.3
Middle temporal gyrus (21)					44	-46	12	4.0*
Cerebellum					6	-64	-22	4.1

The MNI-coordinates and T-values of local maxima within significant clusters are reported. Initial threshold was set at voxel-level p<0.001 uncorrected, with an extended voxel threshold (k) of 8 voxels. Clusters that survived correction for the whole brain volume (p<0.05, FDR-corrected) were considered statistically significant. In addition, we report regions (*) that reached an uncorrected cluster-level significance (p<0.05). Positive x, y and z coordinates indicate respectively coordinates right, anterior and superior of the middle of the anterior commissure. VMcon = visuomotor congruence. VMconSz = visuomotor congruence with different sizes. BA = Brodmann area. MNI = Montreal Neurological Institute. PMd = dorsal premotor cortex. PMv = ventral premotor cortex. pre-SMA = pre-supplementary motor area.

While in both VMcon and VMconSz drawing fully matched the visual template, VMcon contrasted to VMconSz particularly represented size constancy in the congruent tasks. This comparison revealed a bilateral pattern of increased activations comprising lateral and dorsolateral extrastriate visual and superior parietal cortical regions (Fig 4D). The activation in LOC, with dominance in the left hemisphere, was highly specific for VMcon (Figs 3 and 5). This contrast further showed VMcon-related activation in the left PMd. Although the right PMd was also identified at the initial threshold of p<0.001 voxel-level uncorrected (Fig 4D), this activation did not reach statistical significance at cluster-level [x24, y-10, z60] (p = 0.20). Furthermore, significant right cerebellum activation was seen. Coordinates of significant activations are further specified in Table 3.

### Functional differentiation within the PMd

Contrasted to baseline, all five visuomotor tasks resulted in bilateral PMd activations, of which the right (ipsilateral) clusters optimally demonstrated that the focus of maximum activation was located just posterior to the vertical traversing the anterior commissure (Figs 3 and 6A). Subsequent comparisons between these visuomotor conditions showed that Minc was related with activation increases in the rostral segments of the PMd, particularly in the right hemisphere (Fig 6A). Contrasted to Vinc, this rostral extension was bilateral. As reported above, Vinc contrasted to VMcon showed an increase in activation of the superior extension of the pre-SMA over the dorsal surface of the right superior frontal gyrus [x14, y10, z64] reaching the superior part of the rostral PMd (Fig 6A). At this location, responses related to Vinc were similar to that of Minc.

## Discussion

The main aim of the present study was to dissociate motor and perceptual components in cerebral circuitry dealing with incongruity between the sizes of presented and drawn figures. These fundamental aspects of object size in both perception and performance were addressed in order to gain further insight in the way the brain organizes visuomotor transformations implicated in visually guided grasping movement. We were indeed able to demonstrate such dissociation that was, however, of a more complex nature than expected. Right-hemisphere activations distributed over the inferior parietal cortex, dlPFC, pre-SMA/anterior cingulate and frontal operculum/anterior insula were consistently associated with the two incongruity conditions, although generally stronger in Minc than in Vinc, while contrasting the two congruity (control) conditions to each other also revealed characteristic differences in activation. E.g., right inferior parietal, bilateral dlPFC and left PMv activations were increased when the size of presented and drawn figures similarly varied, while copying figures with invariable size showed a characteristic pattern of activations comprising lateral and dorsolateral extrastriate visual areas, superior parietal cortex and caudal segments of the PMd, representing classical nodes in a dorsal visual pathway. As activation of the rostral extension of the right PMd (pre-PMd) was seen in Minc, this indicated a functional dissociation between rostral and caudal segments of the PMd associated with different levels of visuomotor complexity. The unique profile of left PMv activations with similar increase in all conditions characterized by variable size of drawing, irrespective congruity or incongruity with the presented figures, further underscored the importance of size itself as a basic parameter in cerebral processing underlying visuomotor transformations.

### Multiple processing steps in visuomotor incongruity of size

Although specificity and local response magnitudes varied, the pattern of activations that was generally associated with the incongruity conditions comprised the inferior parietal cortex, dlPFC, pre-PMd, pre-SMA/anterior cingulate and frontal operculum/anterior insula, with a right-hemisphere dominance. While our previous functional imaging study on visuomotor incongruity between (one-dimensional) axial orientations demonstrated crucial involvement of the PMd and superior parietal cortex [24], now rostral PMd activation was seen together with activations in more ventral frontoparietal circuitry. This need not be at odds with each other. A combination of PMd—postero-superior parietal activations together with a pattern of dlPFC, inferior parietal, anterior insula and pre-SMA activations has been demonstrated by Cieslik and co-workers using a stimulus-response paradigm with 200ms visual hemifield stimuli followed by motor responses of either the ipsilateral (congruent) or contralateral (incongruent) hand [33]. They argued that the dorsal parietal-premotor regions were implicated in both bottom-up and top-down processing while the ventral frontoparietal activations reflected top-down control mechanisms mediating contextual task demands. Similarly, a distinction between inferior and superior parietal functions has been described concerning action planning and–control, respectively [34]. Consistent with these explanations, particularly the inferior parietal and dlPFC activations in our study indicate that solving visuomotor incongruity concerning figure size implies a planning complexity requiring more intermediate processing steps than needed for solving incongruent axial orientations. The dlPFC and inferior parietal cortex play central roles in cognitive functions such as working memory, attention shifting and response inhibition [35–38], which provides an argument to assume common neuronal mechanisms implicated in visuomotor incongruity and these cognitive functions. The general involvement of these two regions in functions with increased complexity may be based on the fact that temporally sustained activity in dlPFC—inferior parietal circuitry provides a functional interface facilitating other brain regions to guide attention, spatial memory and motor planning [38].

Conditions Mm and Vinc required that the initially observed instruction figure had to be kept in working memory. In Minc, however, the right inferior parietal and dlPFC activations were even stronger than in Mm and Vinc. Considering involvement of these regions in working memory, this suggests that such memory was balanced by more 'covert' working memory in Minc while other cognitive functions might additionally be involved. The involvement of covert working memory would fit the model of visuomotor transformations according which visual information is not directly aligned with a coordinate frame for motor planning, but that visual and motor coordinate frames are separately matched onto an internal frame of reference [24,39]. This multiplicity provides the argument to propose that in Minc, the condition in which actually presented figures were used to make copies with another size, visuomotor transformation is mediated by the cerebral construction of a 'resized' virtual template to serve drawing the instructed size. In this, covert working memory holds the resized template online while the observed figure still provides feature details during drawing. This model of an internal resized template further illustrates that incongruity of object size concerns a higher level of complexity than visuomotor incongruity between basic line orientations. In both, a distinction between perceptual and motor alignment with an internal coordinate system is plausible. However, for visuomotor transformations with incongruent (one-dimensional) linear orientations, without the intermediate processing steps of step-wise matching with a virtual object template, the dissociation between perceptual and motor alignments is achieved within the most dorsal pathway directly interconnecting posterior superior parietal regions and the PMd [24].

### Attention dynamics and inhibitory control

In the previous paragraphs, emphasis was laid on explaining the coherent right inferior parietal and dlPFC activations. In addition, right-hemisphere activations in pre-SMA / anterior cingulate and the anterior insula were consistently seen in Minc and Vinc. While a general involvement of these cortical regions in attention processes and inhibitory control has been described for all four regions, strong functional connections between particularly the anterior insula and the dACC have been described, dissociated from a dlPFC—parietal network [40–42]. In this respect, the latter might serve as a spatial workspace, facilitating anterior insula—pre-SMA/dACC circuitry to exert functions of attention and inhibition to solve visuomotor incongruity in our tasks. A functional distinction that would be consistent with the above quoted model of Ikkai and Curtis [38].

An argument supporting functional coherence between particularly the pre-SMA and anterior insula in our results may be inferred from the fact that the right anterior insula and the most dorsal aspect of the right pre-SMA were the only regions that showed a common activation increase in Minc and Vinc relative to all other conditions. While incongruity of size between the observed figures and (unseen) drawings implied an inhibitory mechanism avoiding an identical copy, image characteristics did serve construction of the same shape. The latter points at a complex balance of 'attention shifting' to both the details of the presented figure and the internal template for resized drawing. Involvement of the anterior insula in such attention dynamics at a short time scale, i.e. recruiting shape details and inhibiting presented size characteristics, seems consistent with its described role in early-stage perceptual decision making [43]. A similar role of particularly the right anterior insula in 'divided' attention has been described during temporal incongruence between visual and auditory stimuli that normally occur synchronously, representing increased perceptual effort to discern small visuo-auditory intervals [44]. In this, Bushara and co-workers pointed at short-latency connections that might particularly enable the insula to mediate early-stage multimodal cortical processing. These characteristics may provide a cue explaining its involvement in a wide range of functions concerning multimodal integration at a basic level, alertness, saliency detection and attention [43,45–52].

In the previous paragraph we pointed at the coherence between pre-SMA and anterior insula activations in our results. In this, pre-SMA activations included parts of the adjacent dACC. The dACC has been strongly implicated in cognitive control of general conflicting conditions, often together with the anterior insula [53–57]. As incongruity of size between the presented pictures and the instructed drawing may be considered to reflect a 'spatial conflict', the right pre-SMA / dACC involvement in our study is consistent with the described contribution to conflict monitoring as well as inhibitory control concerning subsequent responses. Regarding the latter, ACC activation related to incongruity adjustments has been associated with increased right dlPFC activity [58], further supporting the above described network function of the right anterior insula, pre-SMA / dACC and right dlPFC in our study.

### Dissociation of perceptual and executional components in visuomotor incongruity

One of the main aims of this study was to dissociate the visual and motor components in dealing with visuomotor incongruity. We found that the motor component dominated the pattern of incongruity-related activations. Although directly contrasting Minc and Vinc showed a segregation between dorsal parietal and ventral parieto-temporal activations, the profile of their effect sizes demonstrated that the Vinc-related temporal increase was particularly due to the absence of relative decreases that were present in the other conditions. This is consistent with the absence of temporal cortex activations in Vinc when contrasted to the baseline conditions of passively viewing the central fixation cross. On the other hand, the association between Vinc and temporal activations, which suggests involvement of the ventral visual pathway [9,59], would be consistent with an enhanced demand on visuoperceptual processing in the condition with variation in size of the presented pictures.

While the Vinc-related parietal activation was centered at the surface of the temporo-parietal junction, Minc was related with both postero-superior and antero-inferior parietal activation increases compared to Vinc. The antero-inferior parietal activation followed the ascending limb of the intraparietal sulcus, i.e. the post-central sulcus, which suggests a specific association with somatosensory processing, particularly proprioception [60–63]. Complementary to the enhanced visuoperceptual demand in Vinc, an increased proprioceptive demand may indeed be assumed in Minc. Without visual feedback, motor performance relies stronger on proprioceptive information. Although visual feedback was absent in all conditions of our experiment, the size variation of drawing in Minc, not matching the size of the presented figures, apparently poses an enhanced demand on proprioceptive processing. The absence of increased activation of the primary sensory cortex underscores that this activation along the post-central sulcus concerned higher-order sensory processing, which is consistent with the balanced design for executed movements and overt proprioceptive feedback.

The combination of Minc-related activations in the superior parietal cortex, along the posterior segment of the intraparietal sulcus, and in the PMd demonstrated that this classical dorsal visuomotor pathway was dominated by the executional component of visuomotor incongruity. This increase of PMd activation in Minc, compared to Vinc, concerned both hemispheres; we did not see the hypothesized dissociation between Minc and Vinc based on opposite effects in the two hemispheres. Particularly the rostral PMd segment in the right hemisphere showed a consistent increase of activation in Minc compared to all other conditions. A common finding in Minc and the other tasks was the robust bilateral PMd activation with a maximum at a more posterior location. This rostro-caudal distinction is consistent with a demarcation between pre-PMd (F7) and PMd (F2), implicated in higher-level and basic visuomotor functions, respectively [64–68].

### Activation related to drawing with variable sizes

A most intriguing observation was the unique response profile of the left PMv showing that similar increases of activation occurred in this cortical region during all conditions that required drawing with variable sizes, irrespective congruence or incongruence with the example figure. Such a relation between left PMv activation and 'size execution' suggests similarity with its contribution to grasping movements [69]. In this, fine-tuned finger movements need to match the shape and size of an object reached for [70]. Recruitment of neuronal activity in the PMv, not only during motor preparation but also by object observation [71] and watching the act of grasping by others [72], underscores the solid embedding of this region in a wide range of functions supporting prehension. The association of our PMv activation with particularly the variation in size of drawing points at the executional aspect of these tasks while, in contrast, repeatedly drawing the same size does not pose the executional demand of continuous size adaptation. The PMv contribution to such performance with scaling size other than in actual grasping movements provides support for the idea that size is a basic parameter in motor control. The profile of activation seen in the left PMv was not found in the left antero-inferior parietal cortex (anterior intraparietal sulcus (AIP)). As the PMv and AIP often exhibit similar contributions to visually guided grasping [1], important differences have been recognized too. Concerning the recordings from motor-dominant neurons, the parietal cortex appears to be stronger involved in the entire hand action, while PMv neurons are more commonly active during a restricted segment of the action [70]. A dissociation between PMv and AIP functions based on a similar principle may also be inferred from the fMRI study of Harpaz et al. [73] who demonstrated that the identity of written letters was encoded in core motor regions such as the primary motor cortex and AIP, regardless the size of such letters, while for the left PMv letter identity was overruled by the effect of size variation. The exclusive involvement of the left PMv in drawing with variable sizes we found may thus add an argument to the strong executional character of this PMv size function.

The strong contribution of the left PMv to size variance in drawing that we inferred from our paradigm complements and may additionally explain the absence of scale-invariant encoding of letter writing in the PMv as reported in the Kadmon Harpaz study [73]. This complementary character of results obtained from different analysis methods illustrates the value of our classical functional imaging paradigm with conditions balanced in such a way that regional activation increases related to a specific function can be extracted. Given this experimental paradigm and the specific hypothesis underlying its design, we refrained from alternative analysis strategies such as e.g. multivoxel pattern analysis as performed by Kadmon Harpaz et al. [73]. Concerning additional analyses might also consider including actual drawing size as a covariate in the analysis of fMRI data. A limitation of our study is, however, the absence of quantified data on drawing output because subjects continuously drew figures on the same paper during the conditions. We were therefore only globally informed about the actual results of copying size or resizing.

### Size Constancy

While activation increases in the two incongruity conditions (compared to the congruity tasks) logically represented increased complexity of visuomotor control, the unexpected differences between the two congruity tasks point at basic aspects of visual and visuomotor processing concerning size and shape. In the previous paragraph, we treated the unique contribution of the left PMv to drawing variable sizes regardless of the task. In a complementary fashion, the response profile of the extrastriate visual cortex area LOC demonstrated that this region was exclusively activated when various figures with the same size were copied (VMcon). This bilateral activation occurred together with activation of the classical dorsal visual pathway comprising dorsolateral visual and superior parietal cortex as well as the left PMd, identified by contrasting VMcon to the congruity condition in which variably sized figures had to be copied (VMconSz). Visual cortex area LOC plays a central role in general object recognition [10,12,74]. In contrast to e.g. the fusiform face area, its role is not restricted to a distinct category of objects. Although the size of perceived objects has been described to have an effect on LOC activation, distinguishing specific objects irrespective their size induces a more robust effect in LOC [75,76]. Its activation in only VMcon thus suggests that size constancy of the various presented figures in this condition provided a strong cue to evoke LOC responses, efficiently fuelling object information into the dorsal visuomotor pathway [77]. The absence of LOC activation in VMconSz, in which the same figures were presented but with variable extensions in the visual field, suggests that the neuronal inference of 'shape' from these retinal images may be less self-evident than subjectively perceived. Indeed, a variable size of the same figure shape implies different distributions of lines and angles over the retina. One may speculate that reordering such image elements in copying the observed figures represents an enhanced complexity of visuomotor transformation (relative to invariant size copying), requiring circuitry comprising the inferior parietal cortex, dlPFC and PMv.

It thus appears plausible to propose a segregation between visuomotor pathways mediating invariant and variable object size. In this model, visuomotor transformations underlying congruent copying with constant size is channeled via visual area LOC to dorsal parietal-premotor circuitry, while variable size copying is particularly embedded in ventral parietal-premotor circuitry. A role of area LOC in maintaining size constancy is consistent with the observations that lesions in this region may lead to distorted size perception in the contralateral hemifield [78–80]. Size constancy in our experiment implied that the actual dimension of the presented figures and copied drawings did not change. In the literature, size constancy generally refers to the perceptual mechanism that the size attributed to an observed object remains the same despite the changed extension of its retinal representation depending on the distance of the perceived object [25]. Such constancy heavily relies on environmental cues concerning depth and distance and supports adequate grasping movements. This convergence of information about environmental space and object dimensions underscores the functional interactions between processing streams in dorsal and ventral parietal-premotor regions. Artificial manipulation of the perceived object-environment relation may induce illusionary disproportions of object size [25–27]. Coherent involvement of LOC and the superior parietal cortex in size constancy which we demonstrated in the present experiment, has also been described in association with the illusionary change of object (line) size by environmental image manipulation [81]. Common activation of these two regions may thus provide support for equivalent neuronal mechanisms underlying actual and contextual size constancy. In a wider perspective, variance of size thus appears to particularly serve the flexible nature of grasping movements mediated by ventral parietal-premotor circuitry, while size constancy reflects the stability of objects in the context of surrounding space represented in a dorsal parietal-premotor network, which is a prerequisite for a purposeful action such as grasping.

## Conclusions

The patterns of task-related activations in the present study specified distinct characteristics of size emphasizing its role as an essential parameter in visuomotor control. Concerning size incongruity, we proposed a model describing a 'resized' virtual template simultaneously employed with the actual observation of details in the example figures, which provided a consistent explanation for the coherent involvement of right-dominant inferior parietal cortex, dlPFC, pre-SMA/anterior cingulate and frontal operculum/anterior insula, representing aspects of involved cognitive mechanisms such as spatial working memory, early-stage attention shifting and inhibitory control. The increase of left PMv activation in all tasks with variable sizes of drawing, either in congruent or incongruent copying, was attributed to a neuronal mechanism also involved in scaling grasping movement to the size of a target object. In a complementary fashion, our findings related to size constancy added insight in the neuronal significance of perceiving object size in the context of surrounding space.

## Abbreviations

PMd: dorsal premotor cortex
PMv: ventral premotor cortex
LOC: lateral occipital cortex
Minc: motor incongruence
Mm: motor memory
VMcon: visuomotor congruence
VMconSz: visuomotor congruence with different sizes
Vinc: visual incongruence
